# Adaptation of a bidirectional crisis and emergency risk communication framework by community-engaged research partnerships in rural Mississippi during the COVID-19 pandemic

**DOI:** 10.1017/cts.2023.15

**Published:** 2023-02-08

**Authors:** Rodney Washington, Jennifer A. Weis, Mauda Monger, Nakeitra Burse, Sandra Carr Melvin, Angela A. Omondi, Abby M. Lohr, Jane W. Njeru, Caroline E. Compretta, Irene G. Sia, Mark L. Wieland

**Affiliations:** 1 School of Population Health, University of Mississippi Medical Center, Jackson, MS, USA; 2 Center for Clinical and Translational Science, Mayo Clinic, Rochester, MN, USA; 3 My Brother’s Keeper, Inc, Ridgeland, MS, USA; 4 Six Dimensions, LLC, Ridgeland, MS, USA; 5 Institute for the Advancement of Minority Health, Jackson, MS, USA; 6 Department of Behavioral and Environmental Health, School of Public Health, College of Health Sciences, Jackson State University, Jackson, MS, USA; 7 Division of Community Internal Medicine, Geriatrics, and Palliative Care, Mayo Clinic, Rochester, MN, USA; 8 Department of Preventive Medicine, University of Mississippi Medical Center, Jackson, MS, USA; 9 Division of Public Health, Infectious Diseases, and Occupational Medicine, Mayo Clinic, Rochester, MN, USA

**Keywords:** Risk communication, COVID-19, community-engaged research, rural health, health disparities

## Abstract

Community engagement is important for reaching populations at risk for health inequities in the coronavirus disease 2019 (COVID-19) pandemic. A community-engaged risk communication intervention implemented by a community-engaged research partnership in Southeast Minnesota to address COVID-19 prevention, testing, and socioeconomic impacts has demonstrated high acceptability, feasibility, perceived efficacy, and sustainability. In this study, we describe the adaptation of the intervention by a community-academic partnership with rural African American populations in three Mississippi counties with high COVID-19 disparities. Intervention reach was assessed by the number of messages delivered by Communication Leaders to members of their social networks. Perceived scalability of the intervention was assessed by the Intervention Scalability Assessment Tool. Bidirectional communication between Communication Leaders and community members within their social networks was used by the partnership to refine messages, meet resource needs, and advise statewide decision-makers. In the first 3 months, more than 8482 individuals were reached in the three counties. The intervention was deemed to be highly scalable by partnership members. Adaptation of a community-engaged pandemic CERC intervention is feasible and scalable, and it has the potential to reduce COVID-19 inequities across heterogeneous populations. This approach may be incorporated into current and future pandemic preparedness policies for community engagement.

## Introduction

Crisis and emergency risk communication (CERC) frameworks have been used to promote public participation in coronavirus 2019 (COVID-19) mitigation efforts [1,2], but equitable implementation depends on reaching those who have been disproportionately affected by COVID-19 disparities. African American and Hispanic populations have experienced disproportionate COVID-19 incidence, hospitalizations, and deaths due to pandemic-associated environmental and socioeconomic factors framed by structural racism [3,4]. Likewise, immigrant and refugee populations are disproportionately susceptible to COVID-19 and its complications because of limited English proficiency, low access to health care, fear of legal repercussions, employment in sectors where remote working is not possible, and crowded living conditions [5]. Taken as a whole, these groups are more likely to have communication gaps [6], compounded by cultural discordance and mistrust of health institutions [7]. These community voices have not been well represented in development and implementation of COVID-19 CERC, leading to reduced agency to address mitigation strategies, thereby contributing to health disparities [8,9]. Furthermore, a high level of community engagement is needed to reach these populations in times of crisis [1]. Thus, community-engaged CERC has the potential to reduce COVID-19 disparities through shared creation and dissemination of public health messages, enhanced connection to existing resources, and incorporation of community voices in regional pandemic mitigation policies [10].

Due to their preexisting long-term relationships, community-engaged research (CEnR) partnerships, characterized by collaboration between community members and academic partners through all phases of research, are uniquely positioned to operationalize pandemic CERC among health disparity populations [11]. Rochester Healthy Community Partnership (RHCP) is an 18-year community-based participatory research partnership that has addressed multiple health equity issues through participatory work with community partners in Southeast Minnesota [12]. In March 2020, RHCP adopted a CERC framework to address COVID-19 prevention, testing, and socioeconomic impacts within health disparity groups in Olmsted County, Minnesota. Bidirectional communication between Communication Leaders (trusted leaders in the community selected by RHCP community partners for their extensive social connections) and their social networks collaborated with the partnership to refine messages, leverage resources, and advise policy makers. Over the first 14 days of the intervention, messages were delivered by 24 Communication Leaders in 6 languages across 9 electronic platforms to 9882 individuals within their networks [13]. Communication Leader feedback resulted in changes to regional policies that simplified testing logistics and improved provision of essential services (e.g., food and housing) [13]. Subsequent work established feasibility, acceptability, reach, and 12-month sustainability of the community-engaged COVID-19 CERC framework as a tailored intervention for immigrant and refugee populations [14].

Through the National Center for Advancing Translational Sciences Clinical and Translational Science Awards Program, the University of Mississippi Medical Center (UMMC) reached out to Mayo Clinic (RHCP’s primary academic partner) to explore the possibility of adapting the RHCP bidirectional CERC framework in the Mississippi Delta region, where African Americans are disproportionately impacted by COVID-19. Therefore, the objective of this case study is to describe adaptation of the CERC intervention by UMMC to address COVID-19 disparities among rural African American populations in the Mississippi Delta.

## Methods

### Setting and Pandemic Context

The Mississippi Delta has long struggled with health inequities, particularly in African American communities, with higher rates of commonly faced health concerns like obesity, diabetes, infant mortality, and lack of health insurance [15–17]. The COVID-19 pandemic exacerbated longstanding structural inequities that were already pronounced for communities of color living in persistent poverty [3,18,19]. The United States Department of Economic Research states that families living in rural Mississippi Delta face “double exposure” due to poverty at the individual and community levels [20].

At the onset of the COVID-19 pandemic, Mississippians saw a surge in cases in rural African American communities. The three counties identified for scaling of the community-engaged pandemic CERC framework included Holmes, Leflore, and Washington Counties (Table 1). The population in each county is more than 70% African American while the poverty rates are in some cases double the state’s average of 20% (Table 1) [21]. These historically disenfranchised communities in the Deep South face several structural and interpersonal factors that limit their engagement with health systems and providers. COVID-19 is a highly infectious disease, but also preventable by following guidance on mask wearing, social distancing, vaccination, and testing practices. Using our understanding of rural communities in the South, our teams determined that residents were more likely to adopt these safety measures when messaging was crafted and delivered in a language and modality that was culturally tailored and resonated with them personally.

**Table 1.** Demographics of selected Mississippi Delta counties

County	Population	% African American*	Poverty rate*
Holmes	17,000	83%	42%
Leflore	28,183	75%	37%
Washington	43,909	72%	34%

* data.census.gov

### Partnership Building and Composition

In the planning phase of the work, weekly conference calls between RHCP and UMMC academic partners were used to guide and vet approaches, strategies, and lessons learned to inform adaptation of the community-engaged CERC model in the Mississippi Delta. The UMMC partners then formed the Core Advisory Group: a collective membership of more than 18 people including UMMC faculty members, community health workers, nonprofit organizations, and stakeholders who engaged in bi-weekly strategic team meetings. In support of the planning phase, members of the Core Advisory Group offered insight into community-level resources and grassroots organizations working in the area to determine which counties could benefit from the CERC model.

The three priority counties were selected by the Core Advisory Board due to their rural isolation and high proportion of African American residents. These counties had a longstanding history of health disparities and concentrated poverty that represented a larger population of residents in the rural Mississippi Delta region. Additionally, these counties were assessed by the Board to have strong existing grassroots partnerships with potential to rapidly operationalize the intervention framework. Once the priority counties were selected, the next phase of the work was to (1) perform an environmental scan of resources, organizations, and community influencers that could be leveraged and (2) broker connections in regions with high mistrust of health institutions. Employing an environmental scan was an intentional strategy to understand who was on the ground in communities already actively engaged in health promotion in the early days of the pandemic. A listing of agencies, organizations, churches, and other partners was created by the Core Advisory Group to facilitate conversations around their COVID-19 activities in order to promote synergy and prevent duplicative efforts.

After reviewing the county resource landscape, three community partners were recruited (one per county) by Core Advisory Group members to support engagement and implementation efforts. These community partners were longtime residents and held a significant level of influence and reach. Their role was to broker new and existing relationships with civic and faith-based organizations for the purpose of adapting and implementing the community-engaged CERC framework.

### Adaptation of the CERC Intervention Framework

The RHCP CERC framework was adapted to reach African Americans in the Mississippi Delta region with communication gaps due to health disparities and low healthcare access or utilization, health literacy, socioeconomic positioning, and broadband Internet access. The CERC framework provided the structure necessary to create community partner networks, disseminate vital COVID-19 information, and answer community residents’ questions in the context of the rural Mississippi Delta. Thus, the CERC intervention fostered community-driven, bi-directional risk communication specifically tailored for each of the three priority counties.

#### Understanding the audience through listening sessions

In the five months leading up to implementation in 2021, a series of listening sessions were held virtually for varying locations across Mississippi. Residents from North, Central, Delta Region, and Coastal Mississippi were invited to participate in a series of video conferences to understand the pandemic’s impact on contrasting demographics. In those conversations, the key questions were as follows: (1) How has COVID-19 impacted your communities? (2) How did residents most commonly receive important information about COVID-19? (3) What/Who did residents feel were the most reliable sources of information? (4) What were examples of rumors or misinformation they had heard about COVID-19 in their communities? (5) Were there any other concerns about COVID-19 prevention they would like to express? (6) Which members of their community would they recommend to be Communication Leaders to deliver trusted messages about COVID-19?

Each session was recorded and transcribed within and across each county to note common themes from residents and consolidated into concise recommendations that would drive approaches to engagement and messaging. These findings were shared with partners and Communication Leaders to ensure they reflected the collective input of all residents.

Listening sessions were not intended to be formal focus groups. Instead, they were designed to be guided by community members through a fluid conversation. This participant-driven approach was especially important in the Mississippi Delta region, where research is often perceived as extractive without benefitting the community. The listening sessions were an important step for building trust between the Core Advisory Group and residents of the three targeted counties [22,23].

#### Recruitment and training of community partners and communication leaders

From the recommendations gathered during listening sessions, we composed a team of 18 community members with considerable influence in their rural spaces. Each county was represented by one Community Partner and five Communication Leaders. All partners and leaders were trained via video conferencing to understand the reporting forms, message dissemination approaches, and wider aspects of community perspectives on COVID-19. All three counties are rural with similar demographics, but each had their own set of cultural differences. Therefore, training was tailored and held individually with each county team to ensure that the intervention met the specific needs of each community [24,25].

The Community Partner held weekly meetings with Communication Leaders where they disseminated the COVID-19 messaging. UMMC program staff attended all initial meetings to ensure that there was fidelity to the model and support was provided as needed to the Communication Leaders. Community Partners held discussions with members of the Core Advisory Group to understand how messaging was received and any concerns that arose.

#### Culturally responsive COVID-19 messaging

There were two components of COVID-19 messaging. First, community and academic partners co-created formal COVID-19 messages for the Communication Leaders to deliver. Message content was informed by (1) the listening sessions (to tailor messaging to community concerns), (2) discussions between the Core Advisory Group, community partners, and Communication Leaders, and (3) the existing library of RHCP COVID-19 messages. Content was generated weekly across three COVID-19 domains: prevention and awareness, testing, and vaccination awareness. Content was distilled into two to three points per message. A graphic designer was enlisted to take the tailored messaging materials and turn them into reader-friendly infographics and social media content, which were housed on the main partnership Facebook page where Communication Leaders could easily like, save, and repost. The saved messages were used by Communication Leaders to engage their networks in ways most appropriate for their communities (e.g., phone conversations, email, text messages, and social media).

The second component of messaging consisted of informal conversations resulting from the formal messages between the Communication Leaders and community residents. To facilitate such conversations, Communication Leaders were educated by UMMC academic partners on COVID-19 prevention guidelines, transmission, and mitigation of socioeconomic impacts through connection with community resources. This allowed them to verbally answer common questions or dispel misinformation around the virus. Communication Leaders were also encouraged to post messages that they created for others to share in their communities.

Weekly meetings were scheduled between Communication Leaders and UMMC academic partners for each county to understand which approaches in dissemination were most effective, assess feedback on the messages distributed, and determine which concerns to target next. A review of reporting forms maintained by each Communication Leader helped to drive the meetings and provide data points for discussion. Communication Leaders found it useful to remain in contact with the UMMC team throughout the week, outside of their scheduled weekly meetings to discuss community concerns and questions in real time.

### Program Reach and Assessment of Scalability

Reach of the intervention was assessed by the number of messages delivered by Communication Leaders as tracked on their reporting forms.

To assess impressions of sustainability, we employed the Intervention Scalability Assessment Tool [26] (ISAT) with community and academic partners from the Core Advisory Group. This questionnaire includes quantitative and qualitative questions and was designed to help teams identify and assess contextual factors that may facilitate or hinder scale-up. The ISAT also can stimulate and systematically guide conversations about the resources necessary to scale up an intervention. Rather than collation of individual survey results, the ISAT consists of a scalability survey where answers are generated by group discussion. We reported the summary data for the following domains: the problem; the intervention; strategic/political context; evidence of effectiveness (perceived effectiveness); intervention costs and benefits; fidelity and adaptation; delivery settings and workforce; implementation infrastructure; and sustainability. All items were scored on a 3-point Likert scale, with 3 indicating highest scalability potential. We also reported on qualitative considerations composed in the ISAT assessment summary by stakeholders.

## Results

### Results of Listening Sessions

Participants reported significant mistrust of government and healthcare institutions relative to the COVID-19 pandemic and messaging. A grassroots approach to health communication with trusted messengers (including clergy) was endorsed (Table 2).

**Table 2.** Qualitative findings from community listening sessions

Question	Summary of findings	Representative quote
Do you think that there is a level of mistrust that is going on with residents in your county?	Lack of trust in state and national leadership	*“There is no trust in the president and governor, and people do not understand how serious this is, young people do not take it serious.”*
What do you think residents need? What does support look like for residents and what can be done to support families?	Dissemination of preventive messages to residents with a special emphasis on older adults who are less likely to engage in social media	*“Senior residents do not have smartphones and access to social media. Even if they do have smartphones, they do not have access to social media. Information should be added on the local radio stations. Seniors should also be provided with accurate information.”*
Is there anyone you trust more than others?	Include clergy/church leaders in preventive messages; Lack of accurate information from government and media sources	*“Ministers and congregation. However, people are in a place of fear and do not trust anyone. There is distrust in every aspect due to misinformation.”*
How do you feel about possible vaccination (asked as vaccines were first rolling out)?	Lack of trust in the vaccine; Misinformation about the vaccine	*“You have to be honest with what is in the vaccine, many people are against the vaccine, and we think we are the guinea pigs. The government should not provide misinformation on what they want to people to hear. They should be honest about the side effects.”*

### Adapted community-engaged CERC Framework

**Fig. 1.**
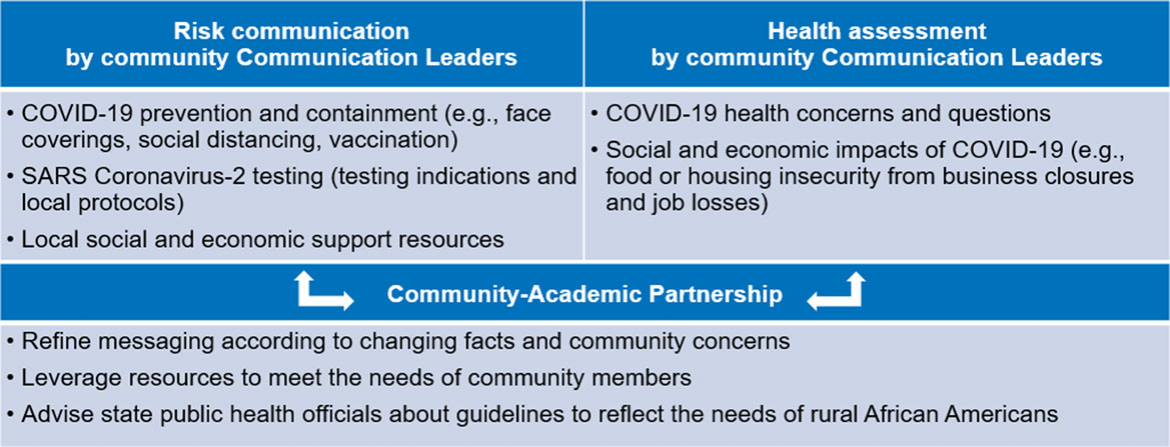
Adapted intervention framework.

UMMC and the Core Advisory Group collaborated with RHCP to adapt the bidirectional community-engaged COVID-19 CERC framework that was community-driven and tailored to each of the three participating counties (Fig. 1).

Each county team worked from a strength-based approach that leveraged existing community assets [27,28]. Communication Leaders disseminated messages in ways that felt comfortable for them. For example, it was not expected that an elder member of the team would circulate content on social media if that was not their preferred platform. However, if phone calls or emails were their strength, they were encouraged to do so.

Context and setting-specific adaptation of the framework (compared with implementation in Minnesota) occurred across each domain as follows:

*Risk communication by Communication Leaders:* The mechanics of risk communication were similar in Minnesota and Mississippi. Namely, dissemination of co-created messages through social networks. However, the nature of social networks varied by site. In Minnesota, social networks were engaged within seven different immigrant and refugee communities. In Mississippi, networks were all within rural African American communities. In Leflore County, communication occurred largely within faith communities. In Holmes and Washington counties, there were more diverse constituents, and social media was used more frequently.

*Health assessment by community Communication Leaders:* The health assessment process in Minnesota and Mississippi was similar in that Communication Leaders solicited COVID-19 concerns and questions from residents.

*Community-Academic Partnership:* The structure of interaction between community and academic partners differed somewhat in Minnesota and Mississippi. In Minnesota, academic partners met regularly with RHCP community partners to refine messages, leverage resources, and develop policy initiatives. The community partners in those meetings then communicated in real time with Communication Leaders. In Mississippi, one member of the academic team (R.W.) met weekly with both the community partners (one per county) and Communication Leaders (five per county) together, with separate meetings for each of the three counties. The community partners then met monthly with the entire Core Advisory Group. In terms of policy, the Minnesota team influenced regional testing and vaccination protocols to make it easier for immigrant and refugee residents to access these services. The Mississippi team influenced statewide policies to lower the threshold for testing among rural African American populations.

### Intervention Reach

Communication Leaders in all three counties used a variety of platforms to share information. While Facebook was the most popular overall, Leflore County also had success disseminating at online workshops. Between March and May 2021, the three Communication Leaders reached 8482 individuals, the majority of whom were in Leflore County (Table 3).

**Table 3.** Number of messages disseminated by Communication Leaders by mode of contact, March–May 2021

Mode of contact	Holmes county	Leflore county	Washington county
Facebook	813	3623	203
Email	2	223	
Messenger	12		28
Twitter	75		
Telephone	12	18	190
Online Worship Service		3283	
Facebook Live	556	22	
Total	1470	7169	421

### Intervention Scalability

The ISAT summary scalability scores by domain are listed in Table 4. The stakeholders concluded that the intervention “merits scale-up.” Scalability was perceived to be facilitated by the magnitude and urgency of the problem, the high perceived efficacy of the intervention that is adapted for local contexts, high acceptability through the tailoring process and co-ownership by community partners, and the relatively low start-up costs of partnering with existing communication leaders in each region. The largest threats to scalability included a lack of normative institutional supports (funding or formal recognition from government and academic partners) for a sustained community-based infrastructure that can react quickly to current and future pandemic needs.

**Table 4.** Scalability constructs of the bidirectional community-engaged pandemic CERC intervention

Domain	Overall score (3-point Likert scale)
The problem	3.0
The intervention	3.0
Strategic/political context	2.5
Evidence of effectiveness	3.0
Intervention costs and benefits	3.0
Fidelity and adaptation	2.5
Reach and acceptability	3.0
Delivery setting and workforce	2.5
Implementation infrastructure	2.5
Sustainability	2.0

## Discussion

This case study described the implementation of an existing community-engaged bidirectional pandemic CERC intervention. The intervention was developed by a community-engaged research partnership with immigrant and refugee populations in Minnesota and adapted by a community-academic partnership with African Americans in the rural Mississippi Delta. While these two populations have differences, such as geographic location and some aspects of their lived experience, they share the burden of being health disparity groups, disproportionately affected by COVID-19. The intervention resulted in dissemination of relevant COVID-19 messages to address local questions while providing a platform to understand issues and barriers directly from community residents, thereby creating a community-driven bidirectional risk communication model specifically tailored for each of the three selected counties with high COVID-19 disparities. The lived experiences of residents were shared with statewide public health decision-makers so that guidelines could reflect the needs of Mississippi Delta residents. In these ways, the intervention is an example of how previously described best practices for pandemic risk communication to historically disenfranchised communities may be applied [29].

This work responds to the call for more research on the process of intervention adaptation in diverse settings to support others in successful adaptation and equitable distribution of evidence-based interventions to address health disparities [30]. Collaboration between UMMC and RHCP community and academic partners resulted in adaptation of the CERC intervention framework for culture and context. It was important that the intervention was adapted by (1) using participatory mechanisms with community partners and (2) tailoring the intervention to address the assets and needs of the priority population

Our findings reenforced the results from two literature reviews. Winograd et al. found that various risk communication interventions that were tailored to specific audiences (e.g., by employing peer educators) consistently demonstrated improvements in cognitive risk perception and intentions to change behavior [31]. Similarly, in a review of adapted psychological interventions for Black and minority populations, Arundell et al. found that adapted interventions were associated with greater symptom improvements posttreatment (compared to non-adapted interventions) [32]. Thus, rather than implementing an intervention with high fidelity, it may be more beneficial to collaborate with the community impacted to adapt interventions, so they are accessible and acceptable to participants.

As part of a national uptake of the RHCP bidirectional community-engaged pandemic CERC model [14,33], this Mississippi case study highlighted the potential scalability of this intervention. The ISAT results indicated that participants viewed the CERC model as meriting scale-up but were concerned about the sustainability of the intervention. These findings emphasize the need and desire for community-based COVID-19 interventions within a healthcare environment with limited resources. To bolster the evidence for the CERC model, going forward, a hybrid effectiveness implementation study using a community-engaged approach would be beneficial [34].

### Implications for Policy and Practice

Public health programming is often created and implemented without community input. The adaptation of the CERC model we describe here is an example of the power of developing an intervention *with* rather than *for* a historically disenfranchised community. Built on community assets – in this case the power of trusted Communication Leaders – the bidirectional flow of communication in this model empowered community members to provide feedback to policy makers during the intense, often fluctuating development of COVID-19 policies and practices. In this way, as a team we centered marginalized voices, adapted a successful intervention that community members would like to see expanded, and responded to community-identified needs. We encourage other public health practitioners and policy makers to consider adapting COVID-19 and future pandemic interventions in collaboration with health disparity populations in ways that uplift community voices and establish and support sustainable mechanisms that address health inequities.

The study has limitations. Dissemination of the messages beyond the initial distribution from Communication Leaders was not quantified, and initial dissemination was under-reported. So, the full intervention reach and amplification effects across social networks cannot be addressed. Although previous research demonstrated that risk communication interventions can be effective at changing perceptions and behaviors [31], risk-related behaviors and outcomes were not assessed in this study. Finally, while the intervention has demonstrated high feasibility among several minoritized populations, community-engaged partnership work is highly contextual, so this process may not be generalizable to some partnerships.

## Conclusions

Adaptation of a community-engaged pandemic CERC intervention is feasible and scalable, and it has the potential to reduce inequities from COVID-19 and future pandemics across heterogeneous populations through shared creation and dissemination of public health messages, deliberate connection to existing resources, and representation of community voices in regional pandemic mitigation policies.
